# Nothing to Do

**Published:** 1866-12

**Authors:** 


NOTHING TO DO.

Every winter the number increases in our large cities, especially in New York, of persons who having become tired of village and country life, or of keeping house in the city, look to boarding at hotels or private houses as a kind of an elysium; nothing to do but eat and sleep and walk about; while there is a blissful deliverence from the annoyances of incompetent, faithless and blarneying servants; who, when you engage them, profess a perfect knowledge and skill in everything which pertains to the kitchen, and yet within a week, show that they know little or nothing beyond the most common duties. They can not even kindle a fire without its costing about three times as much as it ought to; they can not bake a loaf of bread fit to eat; their pastry would kill a plowman in a week; and even when furnished most lavishly with the very best of materials, have neither sense nor system in their preperation; very few know even how to cook a potatoe—and dirt and filth everywhere prevail. But in escaping these vexations, greater than these soon present themselves in a boarding-house life, and among the first is the giving way of the health; the body becomes sleepy and dull; the mind loses its elasticity, and soon begins to waste itself in worriment about the merest trifles; a very little inconvenience or difficulty is magnified and looms up in mountainous proportions. One of these called on us recently, a lady, well educated, dressed elegantly and in perfect taste, and merely to look at, any man might be proud to call her his wife. The moment she entered our office she began to whine at an amazing rate, declaring that she was in a state of perfect physical and mental prostration. It was very natural for us to inquire into the cause of such a hopeless condition. She was boarding. A generous husband had surrounded her with everything calculated to afford her enjoyment. She had no children, and no servants to annoy her. Her husband was at his place of business from 10 to 3, and the remainder of his time was at her service. She went to the races, she attended the opera, and she visited her friends; and yet she pretended she was in a perfect state of physical and mental prostration!

